# Phytochemical Composition and Biological Activity of the Essential Oil from *Ericameria nauseosa* Collected in Southwestern Montana, United States

**DOI:** 10.3390/plants13152063

**Published:** 2024-07-26

**Authors:** Igor A. Schepetkin, Gulmira Özek, Temel Özek, Liliya N. Kirpotina, Andrei I. Khlebnikov, Kevser Ayçiçek, Matthew Lavin, Mark T. Quinn

**Affiliations:** 1 Department of Microbiology and Cell Biology, Montana State University, Bozeman, MT 59717, USA; igor@montana.edu (I.A.S.); lkirpotina@gmail.com (L.N.K.); 2 Department of Pharmacognosy, Faculty of Pharmacy, Anadolu University, Eskisehir 26470, Türkiye; gulmiraozek@gmail.com (G.Ö.); temelozek@gmail.com (T.Ö.); kevseraycicek059@gmail.com (K.A.); 3 Kizhner Research Center, National Research Tomsk Polytechnic University, Tomsk 634050, Russia; aikhl@chem.org.ru; 4 Department of Plant Sciences and Plant Pathology, Montana State University, Bozeman, MT 59717, USA; mlavin@montana.edu

**Keywords:** neutrophil, calcium influx, chemotaxis, immunomodulatory activity, cluster analysis, principal component analysis

## Abstract

*Ericameria nauseosa* (Pall. ex Pursh) G.L. Nesom & G.I. Baird) is used in traditional medicine to treat various diseases; however, little is known about the immunomodulatory activity of essential oil from this plant. Thus, we isolated essential oil from the aerial parts of *E. nauseosa* and evaluated their chemical composition and biological activity. Compositional analysis of *E. nauseosa* essential oil revealed that the main (>2%) components were γ-decalactone (13.3%), cryptone (9.4%), terpinen-4-ol (9.3%), (*E*)-methyl cinnamate (6.0%), T-cadinol (4.7%), spathulenol (3.6%), 8*Z*-2,3-dihydromatricaria ester (3.1%), β-phellandrene (3.0%), *p*-cymen-8-ol (2.2%), 3-ethoxy-2-cycloocten-1-one (2.2%), and *trans*-*p*-menth-2-en-1-ol (2.1%). Distinctive features were the lactones (up to 15%) and polyacetylenes (up to 3.1%), including (2*Z*,8*Z*)-matricaria ester and *8Z*-2,3-dihydromatricaria ester. A comparison with other reported *E. nauseosa* essential oil samples showed that our samples were distinct from those collected in other areas of the country; however, they did have the most similarity to one sample collected in North Central Utah. Pharmacological studies showed that *E. nauseosa* essential oil activated human neutrophil Ca^2+^ influx, which desensitized these cells to subsequent agonist-induced functional responses. Based on our previously reported data that nerolidol, β-pinene, spathulenol, sabinene, and γ-terpinene were active in human neutrophils, these compounds are the most likely constituents contributing to this immunomodulatory activity. However, the relatively high amount of polyacetylenes may also contribute, as these compounds have been characterized as potent immunomodulators.

## 1. Introduction

The Asteraceae (Compositae), commonly known as the sunflower or daisy family, is one of the most species-diverse families of all extant angiosperm families. With an estimated 25,000–35,000 species, this family comprises 10% of all flowering plant species and is distributed worldwide [1,2]. *Ericameria nauseosa* (Pall. ex Pursh) G.L. Nesom & G.I. Baird (also called rubber rabbitbrush) is one of the more common Asteraceae members of the western North American flora. This perennial shrub is characterized by narrow resin-coated greenish-gray leaves, and the leaves and rubbery stems have a soft felt-like covering [3]. *E. nauseosa* yellow flowers bloom from late July to October.

The genus *Ericameria* Nutt. is taxonomically complex and has been historically of interest because of the close phylogenetic relationship between *Ericameria* and the genus *Chrysothamnus* Nutt. and the occurrence of intergeneric hybrids [4]. Several different classifications have been proposed for *Ericameria*, with the most inclusive taxonomical evaluation of the species being published by Hall in 1928 [5]. Since that time, the genus *Ericameria* has been greatly expanded by transfer of species from the genus *Chrysothamnus* and now contains four sections: sect. *Asiris*, sect. *Ericameria*, sect. *Macronema*, and sect. *Stenotopsis* [6]. One of the more recent transfers was *Chrysothamnus nauseosus* (Pall. ex Pursh) Britton, which is now known as *E. nauseosa* (Pall. ex Pursh) G.L. Nesom & G.I. Baird from the section *Macronema* [3,7].

*Ericameria* species have been used ethnopharmacologically throughout history. In reviewing the literature, one must consider reports regarding the use of different *Ericameria* species as well as *Chrysothamnus* species due to their phylogenetic relationship [8]. These plant species produce a considerable variety of secondary metabolites, including grindelane diterpenoids [9], labdane-type acids [10], flavonoids [11], coumarins, phenolic acids, and cinnamic acid derivatives [12], chromanones and acetophenones [13], polyacetylenes [14], and mono- and sesquiterpenes [15]. Although there is little information regarding the biological activity and pharmacological properties of *E. nauseosa*, this species has been used historically by American Indians for a number of medical treatments. For example, the Shoshone Indians used steeped *E. nauseosa* leaves as a tea for colds, coughs, and stomach disorders, and also steeped dried *E. nauseosa* flowers and/or leaves as a general tonic [16]. Likewise, the Northern Cheyenne Indians of Montana used *E. nauseosa* as a remedy for colds, coughs, and tuberculosis [17], and Indian Tribes of Nevada boiled *E. nauseosa* roots and tops together for treating hematochezia [18]. More recently, several studies have investigated the pharmacological properties of some components of *E. nauseosa.* For example, a distillate of *E. nauseosa* was reported to have significant antimicrobial activity [19]. Similarly, *E. nauseosa* leaf extracts were found to have anthelmintic activity [20]. Recently, Hell et al. [11] reported that flavonoids from *E. nauseosa* inhibited the phosphoinositide-3-kinase (PI3K)/protein kinase B (AKT) pathway in human melanoma cells and suggested that they may have potential as anticancer drugs [11].

Among the pharmacologically important bioactive components present in medicinal plant extracts are essential oils, and essential oils have been shown to exhibit immunomodulatory, antimicrobial, antioxidant, and anti-inflammatory effects [21,22,23,24]. Although not much has been reported regarding pharmacological properties of essential oils extracted from *Ericameria* and *Chrysothamnus* species, the sesquiterpene chrysothol isolated from the essential oil of *C. viscidiflorus* Nutt. var. *viscidiflorus* was found to exhibit anti-cancer activity against human breast cancer cells [12]. Essential oil from *C. nauseosus* (Pall.) Britt. var. *nauseosus* has been reported to have antifungal activity against the plant pathogens *Colletotrichum acutatum, C. fragariae*, and *C. gloeosporioides* [25]. However, there is no reported information regarding the pharmacological activity of essential oil from *E. nauseosa*.

We isolated essential oil from the aerial parts of *E. nauseosa* collected in Southwestern Montana and analyzed its chemical composition and biological activity. A comparison with previous reports on *E. nauseosa* essential oils collected in other parts of the country suggested similar and unique components, indicating that the location where these plants are collected affects their essential oil composition. Analysis of its pharmacological properties showed that essential oil isolated from *E. nauseosa* aerial parts was immunomodulatory and activated human neutrophils, leading to downregulated responses to subsequent activation by an inflammatory stimulus. Thus, these studies suggest that essential oils may contribute to the beneficial medicinal properties reported for extracts from *E. nauseosa*.

## 2. Results and Discussion

### 2.1. Composition of Essential Oil from E. nauseosa

Hydrodistillation of *E. nauseosa* aerial parts resulted in a yield of 1.3% (*w*/*v*) essential oil. Gas-chromatography (GC-FID and GC/MS) was used to investigate the essential oil chemical composition, and a total of 74 compounds representing 91.9% of the essential oil were identified and quantified (Table 1). *E. nauseosa* essential oil was predominantly enriched in monoterpenes (oxygenated monoterpenes 37.7%; hydrocarbons 10.0%), with the irregular monoterpene cryptone (9.4%) as the main constituent (Table 2). Among the other important compounds were terpinen-4-ol (9.3%), β-phellandrene (3.0%), *p*-cymen-8-ol (2.2%), *trans*-*p*-menth-2-en-1-ol (2.1%), and *cis-p*-menth-2-en-1-ol (2.0%). This composition was distinguished from those reported previously for *E. nauseosa*/*C. nauseousus*, especially with respect to their high cryptone and terpinen-4-ol contents. Specifically, the major constituents of *C. nauseousus* var. *albicaulis* were β-pinene (16.8%), limonene (18.6%), and β-phellandrene (26.0%); the main constituents of *C. nauseousus* var. *consimilis* were limonene (33.2%), β-phellandrene (18.0%), and (*Z*)-β-ocimene (14.6%); and the major constituents of *C. nauseousus* var. *glabratus* were β-pinene (30.3%), myrcene (10.5%), limonene (16.5%), and β-phellandrene (10.9%) [26]. Finally, essential oils extracted from *E. nauseosa* collected in North Central Utah and Southwestern Idaho were recently reported to contain high percentages of monoterpenes, including β-phellandrene (1.8–56.5%), β-pinene (0.3–23.3%), limonene (0.7–22.3%), and (*Z*)-β-ocimene (0.0–29.3%) [27].

It should be noted that the sesquiterpenes of *E. nauseosa* essential oil were represented only by oxygenated compounds (14.7%), with T-cadinol (4.7%), spathulenol (3.6%), and α-cadinol (1.6%) as the main representatives. In comparison, the sesquiterpene-rich essential oil of *C. nauseosus* ssp. *hololeucus* (A. Gray) H.M.Hall & Clem. collected from a site in Provo, Utah contained (*E*)-β-farnesene (3.4–23.7%), α-muurolene (1.2–7.1%), γ-muurolene (0.9–9.8%), and β-humulene (2.0–3.9%) as the major constituents [48]. Likewise, essential oil of *C. nauseosus* (Pall.) Britt. var. *nauseosus* collected from Blaine County, Idaho (Crockett NW16) contained mono- and sesquiterpenes as the main constituents: β-phellandrene (22.8%), β-pinene (19.8%), and β-eudesmol (7.7%) [25].

A distinctive feature of *E. nauseosa* essential oil was the presence of high concentrations of lactones (15.0%), including γ-decalactone (13.3%) and γ-dodecalactone (1.7%). This is the first time such high amounts of lactones were detected in *Ericameria* or *Chrysothamnus* essential oil. Southwestern Montana *E. nauseosa* essential oil also contained significant amounts of polyacetylenes, including (2*Z*,8*Z*)-matricaria ester and 8*Z*-2,3-dihydromatricaria ester (together 3.1%). These findings are consistent with a previous report by Rose [14], who found that the volatiles of *C. nauseosus* contained methyl *Z,Z*-10-acetoxymatricariate, methyl *Z,Z*-10-hydroxymatricariate, methyl 2(*Z*)-10-acetoxy-8,9-epoxydecen-4,6-diynoate, and methyl 2(*Z*)-10-hydroxy-8,9-epoxydecen-4,6-diynoate and Stirling et al. [27], who reported (*E*,*Z*)- and (*Z*,*E*)-matricaria esters (up to 2.6%) in most essential oils from samples of *E. nauseosa* collected in North Central Utah, but not in Southwestern Idaho.

Another distinguishing property of Southwestern Montana *E. nauseosa* essential oil was the significant content of *cis*- and *trans*-cinnamic acid methyl esters (6.0%). In comparison, essential oils of *E. nauseosa* from Utah [27] contained scarce amounts of cinnamic acid methyl and ethyl esters.

**Figure 1:**
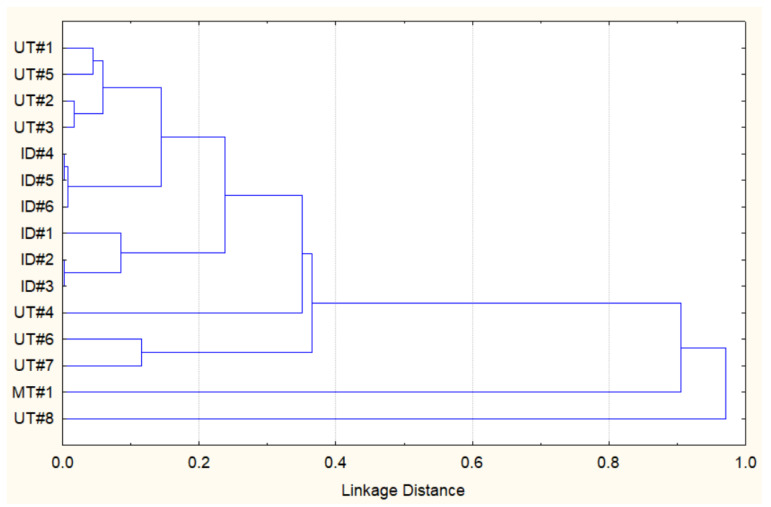
HCA dendrogram representing the similarities of the essential oil compositions of *E. nauseosa* collected in Southwestern Montana (MT#1), North Central Utah (UT#1…8), and Southwestern Idaho (ID#1…6). Pearson correlation was used to measure the linkage distance.

Considering the data above, it appears that the essential oil composition of Southwestern Montana *E. nauseosa* is qualitatively different from those reported previously. However, to further evaluate relationships between the Southwestern Montana *E. nauseosa* essential oil and those previously reported from plants collected in Utah (UT#1…8) and Idaho (ID#1…6) [27], we performed a similar hierarchical cluster analysis (HCA) based on the concentration data for 68 different components (Supplemental Table S1). Figure 1 shows that MT#1 from plants collected in Southwestern Montana and UT#8 from plants collected in North Central Utah seem to be quite similar to each other, whereas they both had low similarities to all of the other essential oil samples.

**Figure 2:**
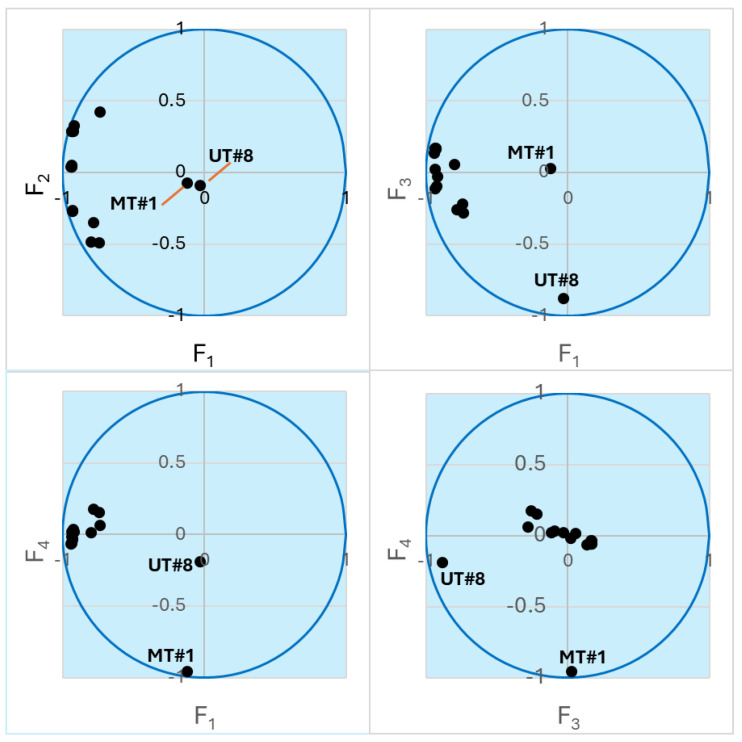
Biplots of the essential oil samples in the axes of the principal components. The points lying close to the unit circle are best described by the corresponding pair of variables. Points for UT#8 and MT#1 are indicated. The other clustered points refer to UT#1…7 and ID#1…6.

To better explore the compound concentration information obtained for the 15 samples (Supplemental Table S1), we performed principal component analysis (PCA), which allows embedding multidimensional data into a subspace of reduced dimensionality defined by mutually orthogonal principal components (PCs). Directions of the principal axes were chosen in order to explain much of the variance in the initial massif of data [49]. Thus, instead of 68 concentration values, each sample can be represented by just a few coordinates in the subspace of the principal components F_1_, F_2_, …, F_n_, which are linear combinations of the actual concentrations. We found that the four most important PCs (F_1_–F_4_) captured 67.92, 8.66, 7.56, and 6.85% of the variance, respectively. In total, 91% of the initial variance was accounted for by these PCs. Thus, the multidimensional initial data can be reduced to four-dimensional subspace without significant loss of information. Note that components F_2_–F_4_ are also of almost equal importance for the data analysis. Figure 2 shows a representation of the samples in biplots of the PCs. The biplots show that the compositions of UT#1…UT#8 and ID#1…ID#6 are well described by components F_1_ and F_2_, while the points for UT#8 and MT#1 lie along the F_3_ and F_4_ axes, respectively, which is indicative of unique compositions of these two essential oils and is in accordance with the HCA results. Nevertheless, the UT#8 datapoint has a noticeable projection along the F_4_ axis towards the MT#1 point (Figure 2). Hence, there may be some similarity between these two samples.

**Figure 3:**
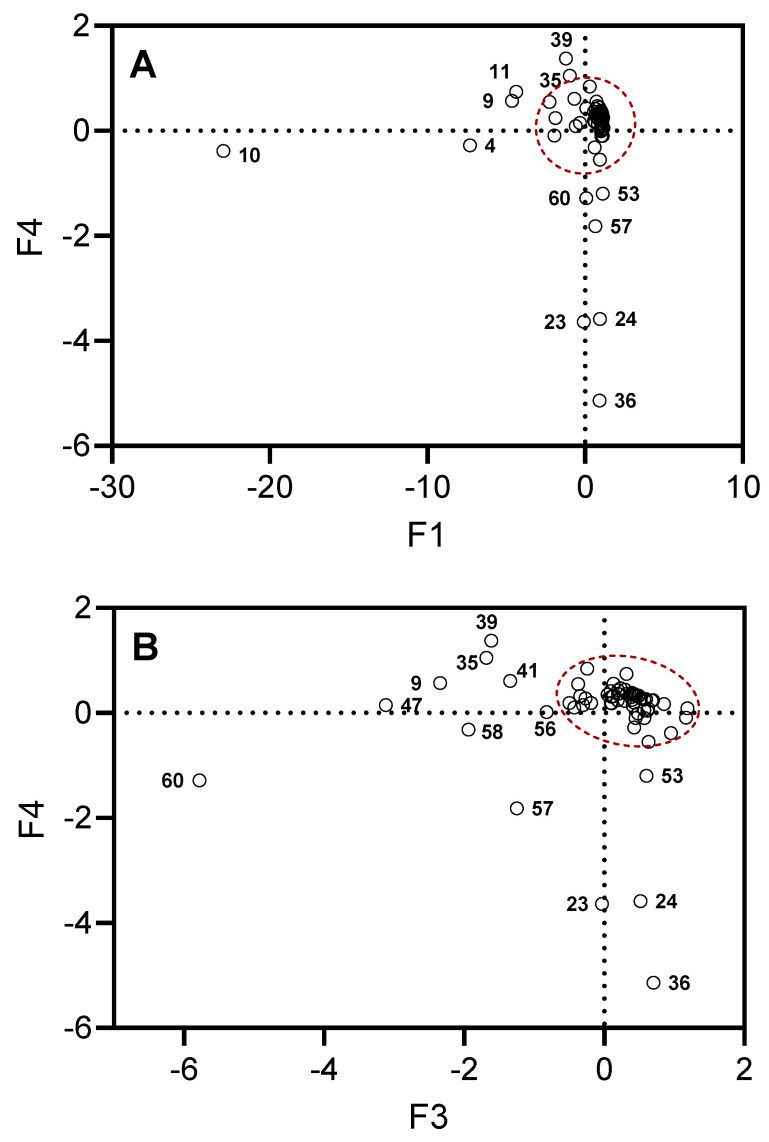
Projections of the selected compounds contained in the essential oils on the planes defined by F_1_, F_4_ (Panel **A**) and F_3_, F_4_ (Panel **B**). The dots outside of the red dashed areas correspond to the compounds most responsible for the differences in MT#1 and UT#8 from the other essential oil samples. Compound numbers correspond to Supplementary Table S1.

Figure 3 shows projections of the selected compounds contained in the essential oils on the planes defined by F_1_, F_4_ and F_3_, F_4_. The projections stretched along the principal axes correspond to the compounds responsible for the key differences between the investigated types of essential oil samples. For example, there is high content of β-phellandrene (compound **10**) in all samples (14.3–56.5%), with the exception of UT#8 (1.8%) and MT#1 (3.0%), and a high content of β-pinene (compound **4**) in all samples (2.2–23.3%), with the exception of UT#8 (0.3%) and MT#1 (1.7%). Although all samples contained γ-curcumene (from traces to 8.3%) and σ-cadinene (from traces to 10.5%), these compounds (**39** and **47**, respectively) were not detected in MT#1. A low amount or even absence (0–3.3%) of terpinen-4-ol (compound **23**), cryptone (compound **24**), and γ-decalactone (compound **36**) was found in all samples, with the exception of MT#1 (9.3%, 9.4%, 13.3%, and 4.7%, respectively). Finally, a low amount or even absence (0–1.2%) of T-cadinol (compound **57**) was found in all samples, with the exception of UT#8 and MT#1. Thus, we conclude that UT#8 and MT#1 are similar but still distinct in their essential oil compositions.

### 2.2. Effect of E. nauseosa Essential Oil on Neutrophil Ca^2+^ Influx and Chemotaxis

**Figure 4:**
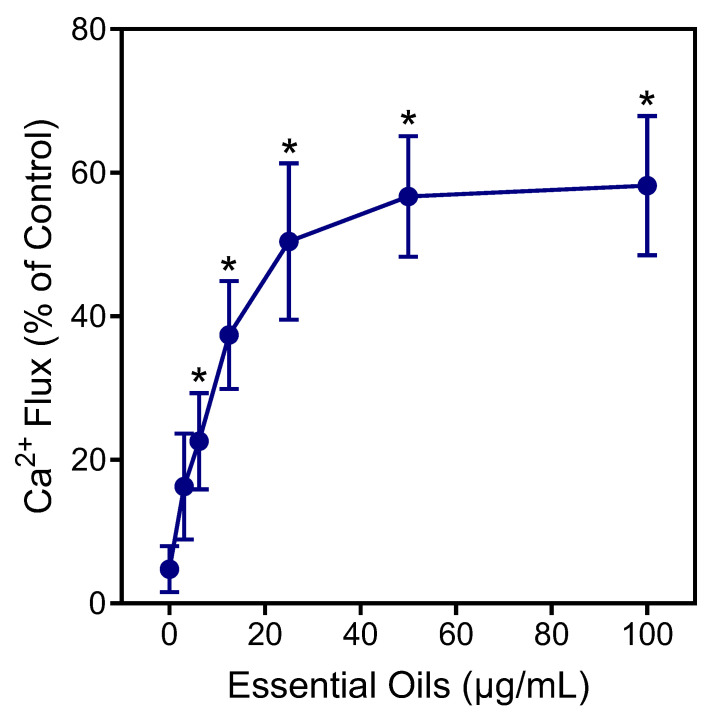
Effect of *E. nauseosa* essential oil on human neutrophil Ca^2+^ mobilization. Human neutrophils were treated with the indicated concentrations of *E. nauseosa* essential oil, and [Ca^2+^]_i_ was measured, as described. The data are expressed as the change in [Ca^2+^]_i_ and compared to control [Ca^2+^]_i_ induced by 5 nM *f*MLF (100%) in neutrophils and plotted as the mean ± SD. The data presented are from one experiment that is representative of three independent experiments with similar results. * *p* < 0.01 compared to dimethyl sulfoxide (DMSO) control [Ca^2+^]_i_.

Neutrophils are essential for host innate immunity and, therefore, represent an ideal pharmacological target for therapeutic development [50]. Thus, we evaluated *E. nauseosa* essential oil for its immunomodulatory effects on human neutrophils. In particular, effects on neutrophil intracellular Ca^2+^ flux ([Ca^2+^]_i_) were evaluated, since [Ca^2+^]_i_ is essential during neutrophil activation [51]. As shown in Figure 4, treatment of neutrophils with *E. nauseosa* essential oil activated these phagocytes, resulting in increased [Ca^2+^]_i_, with an EC_50_ of 27.2 ± 1.4 µg/mL.

**Figure 5:**
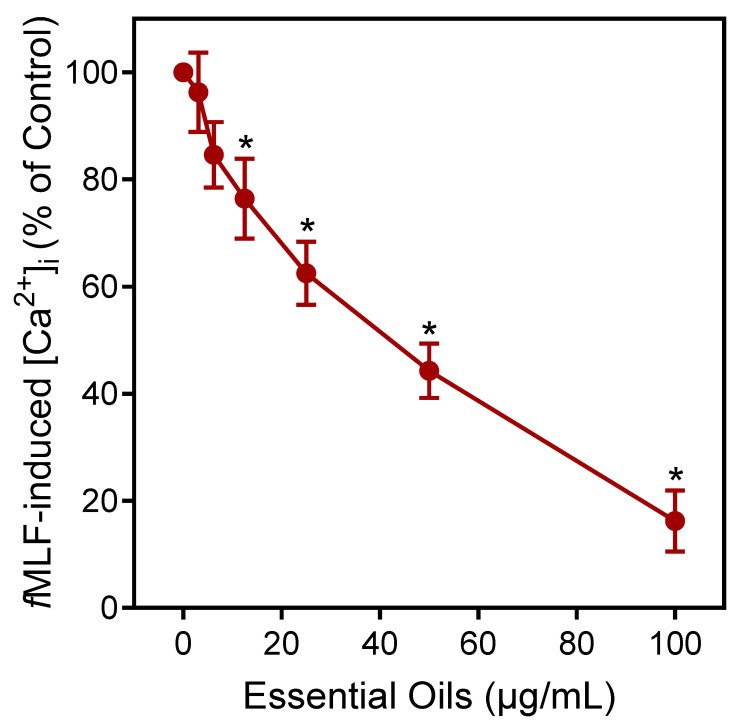
Downregulation of *f*MLF-induced neutrophil Ca^2+^ flux by *E. nauseosa* essential oil. Human neutrophils were pretreated with *E. nauseosa* essential oil or control 1% DMSO for 10 min. After pretreatment, the neutrophils were stimulated with 5 nM *f*MLF, and intracellular Ca^2+^ flux was assessed. The data shown represent the mean ± SD from one experiment that is representative of three independent experiments with similar results. * *p* < 0.01 compared to DMSO control [Ca^2+^]_i_.

Agonists can downregulate or desensitize phagocyte responses to subsequent treatment with homologous or heterologous agonists [52]. Thus, we evaluated whether *E. nauseosa* essential oil could alter agonist-induced [Ca^2+^]_i_ in human neutrophils stimulated with the inflammatory chemoattractant *N*-formyl-methionine-leucine-phenylalanine (*f*MLF). As shown in Figure 5, pre-incubation of neutrophils with *E. nauseosa* essential oil inhibited the subsequent neutrophil [Ca^2+^]_i_ response to *f*MLF with an IC_50_ of 42.1 ± 2.8 µg/mL. Thus, these data confirm that one or more *E. nauseosa* essential oil components can act as human neutrophil agonists and, if briefly encountered by neutrophils, can downregulate neutrophil activation by subsequent agonist treatment.

**Figure 6:**
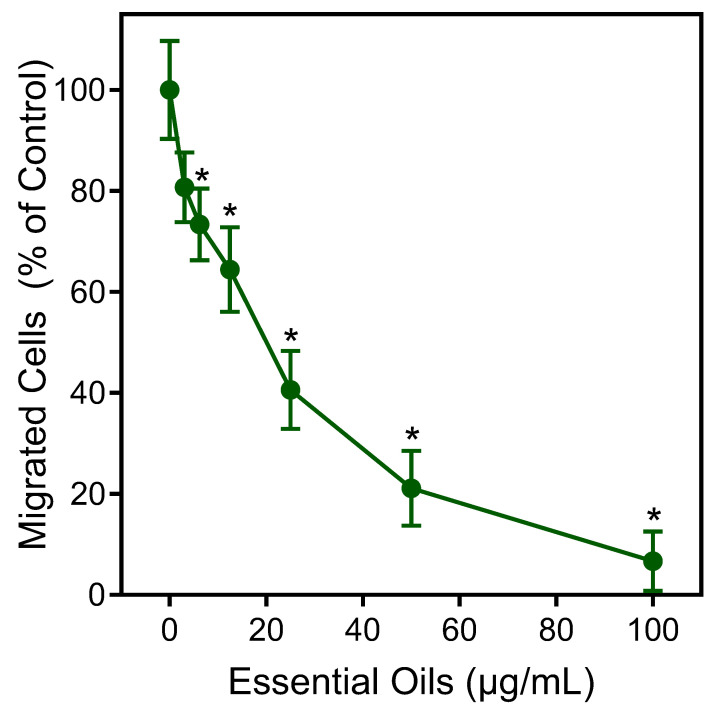
Inhibition of neutrophil chemotaxis by *E. nauseosa* essential oil. Human neutrophils were incubated with *E. nauseosa* essential oil or control 1% DMSO for 30 min. After pretreatment, neutrophil chemotaxis was analyzed using 1 nM *f*MLF as the chemoattractant. The data shown represent the mean ± SD from one experiment that is representative of two independent experiments. * *p* < 0.01 compared to DMSO control [Ca^2+^]_i_.

The effects of the *E. nauseosa* essential oil on human neutrophil chemotaxis were also evaluated. As shown in Figure 6, pretreatment of neutrophils with *E. nauseosa* essential oil dose-dependently inhibited *f*MLF-induced chemotaxis with an IC_50_ of 21.5 ± 3.8 μg/mL, again supporting the conclusion that exposure to *E. nauseosa* essential oil components can downregulate or desensitize neutrophil activation by subsequent agonist treatment.

**Figure 7:**
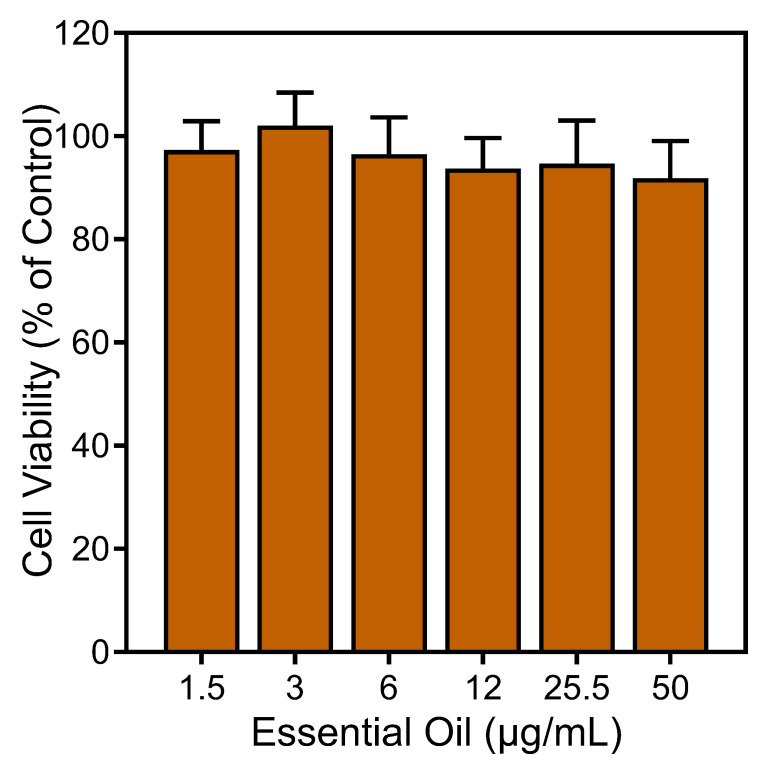
Analysis of the cytotoxicity of *E. nauseosa* essential oil. Human neutrophils were treated with the indicated concentrations of *E. nauseosa* essential oil for 90 min, and cell viability was analyzed, as described. Values are the mean ± SD of triplicate samples from one experiment that is representative of two independent experiments with similar results.

To ensure that the effects of the *E. nauseosa* essential oil on neutrophil responses were not impacted by possible toxicity, we assessed cytotoxicity of our samples (up to 50 µg/mL) during a 90 min incubation period. This incubation period is comparable to the times used in the Ca^2+^ mobilization (up to 30 min) and cell migration (up to 90 min) assays. We found that the *E. nauseosa* essential oil had minimal effects on cell viability during a 90 min incubation, verifying the lack of cytotoxicity during our assays (Figure 7).

In previous studies, we found that *p*-cymene, *p*-cymen-8-ol, limonene, myrcene, (*E*/*Z*)-β-ocimene, β-phellandrene, α-pinene, piperitenone, terpinen-4-ol, α-terpineol, α-thujone, terpinolene, and pulegone did not directly activate human neutrophil [Ca^2+^]_i_ [39,53,54,55,56]. Thus, we can conclude that these compounds, which represent 19.3% of the total composition of *E. nauseosa* essential oil, are similarly not contributing to the neutrophil immunomodulatory activity of this essential oil. In contrast, we reported previously that nerolidol, β-pinene, spathulenol, sabinene, and γ-terpinene activated human neutrophil [Ca^2+^]_i_ [53,55,57]. Thus, these compounds, which represent 6.2% of total composition of *E. nauseosa* essential oil, are likely contributing at least part of its immunomodulatory activity by activating neutrophil Ca^2+^ flux and desensitizing these cells to subsequent agonist activation. Note that ~60% of the remaining components of the *E. nauseosa* essential oil, including polyacetylenes, are not commercially available, have not yet been studied for immunomodulatory activity, and thus may also contribute to the effects on neutrophil function.

**Figure 8:**
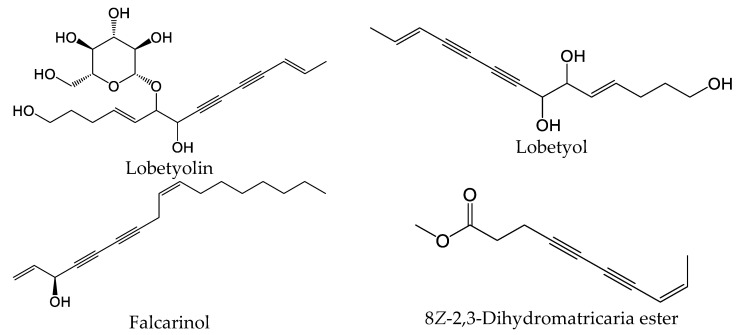
Chemical structures of 8*Z*-2,3-dihydromatricaria ester found in Southwestern Montana *E. nauseosa* essential oil and selected other bioactive polyacetylenes.

Polyacetylenes are a type of compound with carbon–carbon triple bonds and are found in many plant species [58]. This core structure is commonly substituted with alkyl groups, allylic alcohols, and esters in a variety of alkene isomer combinations. Dehydromatricaria-type esters were previously isolated from various plants, including *Artemisia ordosica* [59], *Solidago altissima* [60], and *Tanacetum falconeri* [61]. Chemical structures of 8*Z*-2,3-dihydromatricaria ester, which was found in our *E. nauseosa* essential oil samples, and some selected bioactive polyacetylenes are shown in Figure 8. It should be noted that natural matricaria-type esters, such as the tiglate and hydroxy esters, possess insecticidal activity, as well as activity against *Mycobacterium tuberculosis* [62]. Importantly, pharmacological studies indicate that polyacetylenes possess multiple biological activities, including immunomodulatory activity [58]. For example, lobetyolin significantly downregulated the expression of interleukin (IL)-6, tumor necrosis factor (TNF), and IL-1β in lipopolysaccharide (LPS)-stimulated peritoneal macrophages [63]. This compound was found to be a potent antagonist of G protein-coupled receptor GPR105, which is highly expressed in human neutrophils and sensitive to monosodium urate crystals [64]. Likewise, falcarinol (also known as carotatoxin or panaxynol) and dendranacetylene A were found to inhibit LPS-induced nitric oxide (NO) production in cultured mouse macrophages [65,66]. Finally, adociacetylene A was found to inhibit endothelial cell–neutrophil leukocyte adhesion in vitro [67]. Thus, it is reasonable to suggest that polyacetylenes may contribute to the biological properties of *E. nauseosa* essential oil; however, future studies will be necessary to assess this issue.

## 3. Materials and Methods

### 3.1. Plant Material

We collected wild plants in August 2020, approximately 4 miles south of Norris, MT, USA (45.536519° N, 111.698579° E); voucher specimen number EN-SW-1. Botanical identification of the plant material was performed in the Department of Plant Sciences and Plant Pathology, Montana State University, Bozeman, MT, USA. Aerial parts were air-dried for 7–10 days at room temperature in the dark prior to hydrodistillation.

### 3.2. Chemicals and Reagents

*n*-Hexane was purchased from Merck (Darmstadt, Germany). A C_8_–C_40_ *n*-alkane standard solution was purchased from Fluka (Buchs, Switzerland). Dimethyl sulfoxide (DMSO), N-formyl-Met-Leu-Phe (*f*MLF), ethylenediaminetetraacetic acid (EDTA), and Histopaque 1077 were purchased from Sigma-Aldrich Chemical Co. (St. Louis, MO, USA). Fluo-4AM was purchased from Invitrogen (Carlsbad, CA, USA). Hanks’ balanced salt solution (HBSS; 0.137 M NaCl, 5.4 mM KCl, 0.25 mM Na_2_HPO_4_, 0.44 mM KH_2_PO_4_, 4.2 mM NaHCO_3_, 5.56 mM glucose, and 10 mM HEPES, pH 7.4) was purchased from Life Technologies (Grand Island, NY, USA). HBSS without Ca^2+^ and Mg^2+^ was designated as HBSS^–^; HBSS containing 1.3 mM CaCl_2_ and 1.0 mM MgSO_4_ was designated as HBSS^+^.

### 3.3. Essential Oil Distillation

Essential oil was extracted by hydrodistillation of air-dried plant material (leaves and flowers) using a Clevenger-type apparatus. We used conditions accepted by the European Pharmacopoeia (European Directorate for the Quality of Medicines, Council of Europe, Strasbourg, France, 2014) to avoid artifacts. Yields of the essential oil were calculated based on the amount of air-dried plant material used. Stock solutions of the essential oil were prepared in DMSO (10 mg/mL) for biological evaluation and in *n*-hexane (10% *w*/*v*) for gas chromatographic analysis.

### 3.4. Gas Chromatography–Flame Ionization Detector (GC-FID) and Gas Chromatography–Mass Spectrometry (GC-MS) Analysis

GC-MS analysis was performed with an Agilent 5975 GC-MSD system (Agilent Technologies, Santa Clara, CA, USA), as reported previously [68]. An Agilent Innowax FSC column (60 m × 0.25 mm, 0.25 μm film thickness) was used with He as the carrier gas (0.8 mL/min). The GC oven temperature was kept at 60 °C for 10 min, increased to 220 °C at a rate of 4 °C/min, kept constant at 220 °C for 10 min, and then increased to 240 °C at a rate of 1 °C/min. The split ratio was adjusted to 40:1, and the injector temperature was 250 °C. MS spectra were monitored at 70 eV with a mass range of 35 to 450 *m*/*z*. GC analysis was carried out using an Agilent 6890N GC system. To obtain the same elution order as with GC-MS, the line was split for FID and MS detectors, and a single injection was performed using the same column and appropriate operational conditions. The FID temperature was 300 °C. The essential oil components were identified by co-injection with standards (whenever possible), which were purchased from commercial sources or isolated from natural sources. In addition, compound identities were confirmed by comparison of their mass spectra with those in the Wiley GC/MS Library (Wiley, NY, USA), MassFinder software 4.0 (Dr. Hochmuth Scientific Consulting, Hamburg, Germany), Adams Library, and NIST Library. Confirmation was also achieved using the in-house “Başer Library of Essential Oil Constituents” database, obtained from chromatographic runs of pure compounds performed with the same equipment and conditions. A C_8_–C_40_ *n*-alkane standard solution (Fluka, Buchs, Switzerland) was used to spike the samples for the determination of relative retention indices (RRIs). Relative percentage amounts of the separated compounds were calculated from the FID chromatograms.

### 3.5. Isolation of Human Neutrophils

For isolation of human neutrophils, blood was collected from healthy donors in accordance with a protocol approved by the Institutional Review Board at Montana State University (protocol #MQ041017). Neutrophils were purified from the blood using dextran sedimentation, followed by Histopaque 1077 gradient separation and hypotonic lysis of red blood cells, as described previously [57].

### 3.6. Ca^2+^ Mobilization Assay

Changes in intracellular Ca^2+^ concentrations ([Ca^2+^]_i_) were measured with a FlexStation 3 scanning fluorometer (Molecular Devices, Sunnyvale, CA, USA), as described previously [57]. To assess the direct effects of pure essential oil on Ca^2+^ influx, the essential oil was added to the wells (final concentration of DMSO was 1%), and changes in fluorescence were monitored (λ_ex_ = 485 nm, λ_em_ = 538 nm) every 5 s for 240 s at room temperature after addition of the test compound. To evaluate inhibitory effects of the compounds on FPR1-dependent Ca^2+^ influx, the compound/oil was added to the wells (final concentration of DMSO was 1%) with human neutrophils. The samples were preincubated for 10 min, followed by addition of 5 nM *f*MLF. The maximum change in fluorescence, expressed in arbitrary units over baseline, was used to determine the agonist response.

### 3.7. Chemotaxis Assay

To evaluate the effects of the *E. nauseosa* essential oil and its components on neutrophil migration, we resuspended the neutrophils in chemotaxis media (HBSS^+^ containing 2% (*v*/*v*) heat-inactivated FBS) at 2 × 10^6^ cells/mL. We analyzed chemotaxis using 96-well ChemoTx chambers (Neuroprobe, Gaithersburg, MD, USA), as described previously [57]. Calculation of median effective concentrations (IC_50_) was performed by nonlinear regression analysis of the dose–response curves.

### 3.8. Cytotoxicity Assay

Cytotoxicity of the essential oil and pure compounds in human neutrophils was analyzed with a CellTiter-Glo Luminescent Cell Viability Assay Kit (Promega, Madison, WI, USA) according to the manufacturer’s protocol and as previously described [57].

### 3.9. Statistical Analysis

The concentrations of 68 compounds found in our essential oil samples from plants collected in Southwestern Montana (MT#1) and those reported for essential oil samples from plants collected in North Central Utah and Southwestern Idaho (UT#1…UT#8, ID#1…ID#6) [27] (see Supplemental Table S1) were used for comparison and statistical analyses. Concentrations indicated as “trace” were assigned as 0.01% for statistical analysis. HCA and principal component analysis (PCA) were performed using STATISTICA 6.0 software (StatSoft, Moscow, Russia). For HCA, the unweighted pair group method with arithmetic average was used for cluster definition, while the Pearson correlation was applied to measure linkage distance or similarity. For performing the PCA, components F_1_–F_4_ (each accounting for >5% of the initial variance) were retained and used to explore the differences in the sample compositions. One-way analysis of variance (ANOVA) was performed on datasets in the biological experiments, followed by Tukey’s pair-wise comparisons. Pair-wise comparisons with differences at *p* < 0.05 were considered to be statistically significant.

## 4. Conclusions

Essential oil isolated from the leaves of *E. nauseosa* contained relatively high (>2%) amounts of γ-decalactone, cryptone, terpinen-4-ol, (*E*)-methyl cinnamate, T-cadinol, spathulenol, 8*Z*-2,3-dihydromatricaria ester, β-phellandrene, *p*-cymen-8-ol, 3-ethoxy-2-cycloocten-1-one, and *trans*-*p*-menth-2-en-1-ol. Overall, *E. nauseosa* essential oil was predominantly composed of monoterpenes (oxygenated monoterpenes and hydrocarbons), with cryptone as the main representative. The sesquiterpenes were represented only by oxygenated compounds, with T-cadinol, spathulenol, and α-cadinol as the main representatives. Distinctive components were the lactones (up to 15%) and polyacetylenes (up to 3.1%), namely (2*Z*,8*Z*)-matricaria ester and *8Z*-2,3-dihydromatricaria ester, which are structurally characterized by a conjugated ene–diyne–ene system. A comparison with other reported *E. nauseosa* essential oil samples showed that our samples from plants collected in Southwestern Montana were quite distinct from those collected in other areas of the country; however, they did have the most similarity to one sample collected in North Central Utah.

Pharmacological studies showed that *E. nauseosa* essential oil activated human neutrophil Ca^2+^ influx, which desensitized these cells to subsequent agonist-induced functional responses. Based on previously reported data that nerolidol, β-pinene, spathulenol, sabinene, and γ-terpinene were active in human neutrophils, these compounds are the most likely constituents contributing to this immunomodulatory activity. However, the relatively high amount of polyacetylenes may also contribute, as these compounds have been characterized as potent immunomodulators. Thus, future studies are needed to evaluate the potential of the matricaria esters as modulators of innate immune cells. In addition, it will be interesting to evaluate potential anti-inflammatory properties of polyacetylene compounds if they really do contribute to the therapeutic effects of *E. nauseosa* essential oil; however, this will require extensive work to isolate these pure compounds.

## Figures and Tables

**Table 1:** Chemical composition of essential oil from *E. nauseosa* of Southwestern Montana.

No	RRI ^a^	RRI ^b^	Compound	% ^c^	No	RRI ^a^	RRI ^b^	Compound	% ^c^
1	1032	1008–1039 [28]	α-Pinene	t	38	1729	1729 [29]	*cis*-1,2-Epoxy-terpin-4-ol	0.2
2	1035	1012–1039 [28]	α-Thujene	t	39	1733	1693–1740 [28]	Neryl acetate	0.2
3	1048	1051 [30]	2-Methyl-3-buten-2-ol	0.3	40	1744	1687–1770 [28]	Phellandral	0.4
4	1118	1085–1130 [28]	β-Pinene	1.7	41	1748	1689–1748 [28]	Piperitone	0.6
5	1132	1098–1140 [28]	Sabinene	0.4	42	1751	1699–1751 [28]	Carvone	0.5
6	1174	1140–1175 [28]	Myrcene	0.1	43	1758	1668–1771 [28]	*cis*-Piperitol	0.9
7	1176	1148–1186 [28]	α-Phellandrene	0.1	44	1786	1743–1788 [28]	*ar*-Curcumene	t
8	1195	1167–1197 [28]	Dehydro-1,8-cineole	t	45	1802	1747–1805 [28]	Cumin aldehyde	0.9
9	1203	1178–1219 [28]	Limonene	1.4	46	1811	1814 [31]	*p*-Mentha-1,3-dien-7-al	0.3
10	1218	1188–1233 [28]	β-Phellandrene	3.0	47	1864	1813–1865 [31]	*p*-Cymen-8-ol	2.2
11	1246	1211–1251 [28]	(*Z*)-β-Ocimene	1.4	48	1912	1912 [25]	*cis*-Dihydrocarveol	0.4
12	1255	1222–1266 [28]	γ-Terpinene	0.1	49	1969	1914–1977 [25]	*cis*-Jasmone	0.8
13	1266	1232–1267 [28]	(*E*)-β-Ocimene	0.4	50	1981	1969 [32]	(*Z*)-Methyl cinnamate	t
14	1280	1246–1291 [28]	*p*-Cymene	0.8	51	1981	1981 [33]	Cuminyl acetate	t
15	1290	1261–1300 [28]	Terpinolene	0.5	52	2023	2065 [34]	*p*-Mentha-1,4-dien-7-ol *	0.9
16	1413	1413 [35]	Rose furan	0.4	53	2030	1961–2033 [28]	Methyl eugenol	0.2
17	1437	1385–1441[28]	α-Thujone	0.5	54	2039	-	1-Hydroxy-pseudodiosphenol	1.3
18	1443	1452 [36]	2,5- Dimethylstyrene	0.1	55	2050	1995–2055 [28]	(*E*)-Nerolidol	0.4
19	1474	1425–1478 [28]	*cis*-Sabinene hydrate	0.4	56	2071	2003–2071 [28]	Humulene epoxide-II	0.1
20	1477	1477 [35]	4,8-Epoxyterpinolene	0.2	57	2096	2046–2105 [28]	(*E*)-Methyl cinnamate	6.0
21	1483	1483 [37]	Octyl acetate	0.1	58	2098	2049–2104 [28]	Globulol	0.3
22	1487	1487 [38]	Isoneroloxide	t	59	2113	2070–2114 [28]	Cumin alcohol	1.5
23	1498	–	(*E*)-β-Ocimene epoxide	0.1	60	2115	2115 [39]	4-Hydroxy-4-methyl-cyclohex-2-enone	0.8
24	1529	1529 [40]	Dill ether	t	61	2144	2074–2150 [28]	Spathulenol	3.6
25	1541	1481–1555 [28]	Benzaldehyde	0.2	62	2170	2090–2189 [28]	β-Bisabolol	1.1
26	1556	1526–1565 [28]	*trans*-Sabinene hydrate	0.5	63	2183	2090–2178 [28]	γ-Decalactone	13.3
27	1571	1557–1625 [28]	*trans-p*-Menth-2-en-1-ol	2.1	64	2187	2184 [41]	*T*-Cadinol	4.7
28	1611	1564–1630 [28]	Terpinen-4-ol	9.3	65	2209	2200 [42]	*T*-Muurolol	0.8
29	1626	1626 [43]	2-Methyl-6-methylene-3,7-octadien-2-ol	t	66	2214	2214 [25]	*ar*-Turmerol	0.4
30	1638	1555–1645 [28]	*cis-p*-Menth-2-en-1-ol	2.0	67	2241		*p*-Isopropyl phenol	0.3
31	1648	1597–1648 [28]	Myrtenal	t	68	2249	2227 [44]	8*Z*-2,3-Dihydromatricaria ester	3.1
32	1655	1670 [45]	Chrysanthenyl isobutyrate	1	69	2255	2180–2255 [28]	α-Cadinol	1.6
33	1662	1626–1663 [28]	Pulegone	0.1	70	2272	-	4-Hydroxycryptone	0.5
34	1678	1620–1678 [28]	*cis-p*-Mentha-2,8-dien-1-ol	0.2	71	2309	2336 [44]	(2*Z*,8*Z*)-Matricaria ester	t
35	1690	1644–1690 [28]	Cryptone	9.4	72	2368	2368 [46]	Eudesma-4(15),7-diene-1-b-ol	0.2
36	1700	1662–1717 [28]	*p*-Mentha-1,8-dien-4-ol	0.8	73	2396	2396 [47]	γ-Dodecalactone	1.7
37	1706	1659–1724 [28]	α-Terpineol	1.9	74	2430	-	3-Ethoxy-2-cycloocten-1-one *	2.2

Legend: Major compounds with abundances >2% are highlighted in bold. RRI ^a^, relative retention index calculated on the basis of retention of *n*-alkanes; RRI ^b^, relative retention index reported in literature; % ^c^, calculated from flame ionization detector data. The data are presented as relative % for each component that was identified in the essential oil; *t*, trace amounts were present at <0.1%; * tentatively identified using Wiley and MassFinder mass spectra libraries and published RRI. All other compounds were identified by comparison with co-injected standards.

**Table 2:** Distribution of major compound classes in *E. nauseosa* essential oil.

Number of Compounds	Class	%
**13**	Monoterpene hydrocarbons	10.0
**34**	Oxygenated monoterpenes	37.7
**1**	Sesquiterpene hydrocarbons	t
**11**	Oxygenated sesquiterpenes	14.7
**2**	Lactones	15.0
**2**	Acetylenic derivatives	3.1
**2**	Benzenoid aromatics	6.0
**9**	Others	5.4

## Data Availability

Data are contained within the article and Supplementary Materials.

## Supplementary Materials

The following supporting information can be downloaded at: https://www.mdpi.com/article/10.3390/plants13152063/s1, Supplementary Table S1. Chemical composition of essential oil from *E. nauseosa* of North-Central Utah (UT#1-8), Southwestern Idaho (ID#1-6), and Southwestern Montana (MT#1). Supplementary Figure S1. GC Chromatogram of *E. nauseosa* essential oil.
