# Supplementary material for: Phytochemical Composition and Biological Activity of the Essential Oil from Ericameria nauseosa Collected in Southwestern Montana, United States
Source: Plants (Basel). 2024 Jul 26;13(15):2063. doi: 10.3390/plants13152063 (PMC11314070; doi:10.3390/plants13152063)
Supplement: Supplementary file 1 [file plants-13-02063-s001.zip › plants-3110254-supplementary.pdf]

Supplementary Materials

for

Phytochemical Composition and Biological Activity of the Essential Oil from *Ericameria nauseosa* Collected in Southwestern Montana, United States

Igor A. Schepetkin[1], Gulmira Özek[2], Temel Özek[2], Liliya N. Kirpotina[1], Andrei I. Khlebnikov[3], Kevser Ayçiçek[2], Matthew Lavin[4], and Mark T. Quinn[1*]

[1]Department of Microbiology and Cell Biology, Montana State University, Bozeman, MT 59717, United States

[2]Department of Pharmacognosy, Faculty of Pharmacy, Anadolu University, Eskisehir 26470, Türkiye

[3]Kizhner Research Center, National Research Tomsk Polytechnic University, Tomsk 634050, Russia

[4]Department of Plant Sciences and Plant Pathology, Montana State University, Bozeman, MT 59717, United States

*Correspondence:

Mark T. Quinn (mquinn@montana.edu); Tel.: +1 406-994-4707

Supplementary Table S1. Chemical composition of essential oil from *E. nauseosa* of North-Central Utah (UT#1-8), Southwestern Idaho (ID#1-6), and Southwestern Montana (MT#1)

#	**Compound**	**UT#1**	**UT#2**	**UT#3**	**UT#4**	**UT#5**	**UT#6**	**UT#7**	**UT#8**	**ID#1**	**ID#2**	**ID#3**	**ID#4**	**ID#5**	**ID#6**	**MT#1**
**1**	α-Thujene	0.3	0.1	0.2	0.2	0.2	tr	0.1		0.2	0.4	0.3	0.2	0.2	0.5	tr
**2**	α-Pinene	1	0.3	0.6	0.8	0.7	0.1	0.2		0.2	0.6	0.5	0.6	0.6	0.6	tr
**3**	Sabinene	7.6	3.6	5.4	1.3	2.6	tr	0.2		2	5.2	6.8	7.5	8.8	5.8	0.4
**4**	β-Pinene	23.3	10	15.2	9	11.3	2.2	3.4	0.3	4.2	13.7	13.4	13	12.6	12.2	1.7
**5**	Myrcene	4.7	10.9	12.9	5.9	7.2	0.8	0.7		1.2	1.3	1.1	1.2	1.5	1.3	0.1
**6**	α-Phellandrene	0.2	0.1	0.2	1.1	1.2	0.6	0.4		0.1	0.3	0.3	0.2	0.2	0.2	0.1
**7**	α-Terpinene	0.7	0.6	0.8	1.1	1.1	0.7	0.7		0.5	0.9	0.8	0.3	0.4	0.9	
**8**	*p*-Cymene		0.1	0.1	0.4	0.2	0.6	0.2		0.1	0.2	0.1	0.1	0.1	tr	0.8
**9**	Limonene	15.4	8.1	8.9	22.3	13.9	5.6	1.7	0.9	5.8	0.7	0.8	0.8	0.8	2.6	1.4
**10**	β-Phellandrene	35.9	25.2	26.3	15.8	26.4	14.4	14.3	1.8	29.4	36.6	37.4	53	48.5	56.5	3
**11**	(*Z*)-β-Ocimene		0.2	0.2	0.1	0.1	12.9	6.8		29.3	19.5	19.5	0.2	0.3	0.3	1.4
**12**	(*E*)-β-Ocimene	3.9	7.7	6.8	5.4	4	0.9	0.5		2.1	4.3	1.9	3.7	4.5	3.9	0.4
**13**	γ-Terpinene	1.1	1	1.1	2.2	1.9	1.6	1.4		0.8	1.5	1.3	0.6	0.7	1.5	0.1
**14**	*cis*-Sabinene hydrate									0.2	0.4	0.5	0.3	0.4	0.7	0.4
**15**	Terpinolene	0.3	0.3	0.3	1.1	0.9	0.6	0.4		0.2	0.5	0.3	0.2	0.2	0.3	0.5
**16**	*trans*-Sabinene hydrate									0.1	0.3	0.4	0.3	0.3	0.7	0.5
**17**	α-Thujone												0.1	0.1		0.5
**18**	Cosmene		0.2		tr		tr	0.1		3.5	0.7	0.6	1.2	1.5	0.6	
**19**	*cis*-*p*-Menth-2-en-1-ol		tr	tr	0.1	0.1	0.2	0.1		0.3	0.7	0.8	0.4	0.6	0.6	2
**20**	*allo*-Ocimene						0.6	0.4		1.9						
**21**	1,3,8-*p*-Menthatriene		1.3	0.7	0.6	0.4	0.1	0.2		4	3.9	4.2	9	7.1	3.8	
**22**	*trans*-*p*-Menth-2-en-1-ol				tr		0.1	0.1		0.2	0.5	0.5	0.3	0.4	0.3	
**23**	Terpinen-4-ol	0.8	0.4	0.3	0.5	1.2	1.4	0.6	3	2.2	3.3	3.2	1.3	1.5	2.7	9.3
**24**	Cryptone				0.1		0.2	0.1	0.6	0.1	0.3	0.1	0.3	0.5	0.1	9.4
**25**	α-Terpineol		0.1	tr	0.1	0.2	0.4	0.2	1.7	0.2	0.4	0.4	0.2	0.2	0.2	
**26**	*cis*-Piperitol				tr		0.2			0.1	0.1	0.2	0.1	0.1	0.1	0.9

**27**	Octyl acetate		0.3	0.2	0.2	0.1	0.5	0.4								
**28**	Phellandral		tr	0.1	0.1	0.1	0.9	0.6			tr	tr	tr	0.1	tr	0.4
**29**	Citronellyl acetate		0.6	0.4	0.5	0.4	0.2	0.2	3.4			tr	tr		tr	
**30**	Geranyl acetate		3.2	2	1.3	0.9	0.4	0.3	3							
**31**	*trans*-Myrtenyl acetate						0.8	0.7								
**32**	7-*epi*-Sesquithujene		0.3	0.2	0.4	0.4	0.6	0.9	0.2	tr						
**33**	*trans*-β-Elemene		0.3	0.2	0.2	0.2		0.1	1.6	0.1						
**34**	Methyleugenol		0.9	0.6	0.3	0.3	0.6	0.4	0.5					tr		0.2
**35**	(*E*)-β-Caryophyllene	1.6	4.1	2.8	1.9	1.7	6.2	7	2.8	tr	tr		tr	0.1	tr	
**36**	γ-Decalactone				0.2	0.3				0.2	tr	tr	0.1	0.2	tr	13.3
**37**	Ethyl (*E*)-cinnamate				0.1		0.9	0.8								
**38**	γ-Muurolene		0.4	0.3	0.6	0.6	0.7	0.7	0.9	0.1					tr	
**39**	γ-Curcumene	0.4	1.7	1	3.4	4.2	8.2	8.3	1	0.1	0.1	tr	0.2	0.2	tr	
**40**	*ar*-Curcumene						6.6	2.9			tr	tr	0.1	0.2	tr	
**41**	Germacrene D	2.2	4.6	2.8	4.5	4.5		5.1	3	0.1						
**42**	α-Zingiberene		0.1		0.1	0.1	0.4	0.8								
**43**	α-Muurolene		0.7	0.6	0.7	0.6	0.8	1	2.2	0.2			tr		tr	
**44**	β-Bisabolene		0.1	tr	0.2	0.2	0.6	0.8					tr	tr	tr	
**45**	β-Curcumene		0.3	0.2	0.5	0.6	1.4	1.5		tr			tr	tr	tr	
**46**	γ-Cadinene		0.8	0.6	0.8	0.9	1.3	1.2	2.2	0.2	tr				tr	
**47**	δ-Cadinene	0.6	2.9	2.3	3.1	2.4	2.8	3.4	10.5	1.4	tr	tr	0.1	0.1	0.1	
**48**	(*E*,*Z*)-Matricaria ester		0.1		0.3	0.1	0.3	0.8								
**49**	(*Z*,*E*)-Matricaria ester		0.1		0.3	0.2	2.6	1.9								
**50**	α-Cadinene		0.1	0.1	0.2	0.1	0.2		0.5	tr						
**51**	α-Elemol									1.5	0.6	1.2	1.3	0.5		
**52**	(*E*)-Nerolidol				0.5	0.3	2	2.6						tr		0.4
**53**	Spathulenol					0.1										3.6
**54**	Caryophyllene oxide		0.3	0.3	0.2	0.1	0.4	1.1	1.1							
**55**	Viridiflorol		0.3	0.2	0.3	0.1	0.4	0.4	2.9							
**56**	α-Oplopenone		0.6	0.4	0.1		0.2	0.4	5							
**57**	τ-Cadinol		0.6	0.4	1.1	0.6	1.1	1.2	5.9	0.4	tr				0.1	4.7
**58**	τ-Muurolol		0.8	0.4	1.1	0.8	1.5	1.5	8.1	0.5	tr				0.1	0.8

**59**	α-Muurolol		0.2	0.1	0.4	0.2	0.4	0.5	2.4	0.2	tr				tr	
**60**	α-Cadinol		1.6	0.8	2.3	1.4	2.5	2.5	21.7	2.1						1.6
**61**	β-Eudesmol										1.7	1.7	2.3	4.1	1.5	
**62**	β-Bisabolol				0.1		0.5	0.8			tr	tr	tr	tr	tr	1.1
**63**	α-Bisabolol				0.2		0.2	0.5								
**64**	Phytone		0.3	0.2	0.2	0.1	0.3	1.5	0.6					tr		
**65**	Ethyl palmitate						0.2	1								
**66**	Phytol				0.3		0.3	1.8	1.1		tr	tr	0.1	0.2	tr	
**67**	Ethyl linolenate						0.3	1.4								
**68**	Tricosane		0.2	0.2	0.2		0.6	1.9			tr			tr		

Data for the components of essential oils from *E. nauseosa* collected in North-Central Utah and Southwestern Idaho are taken from Stirling et al. [27].

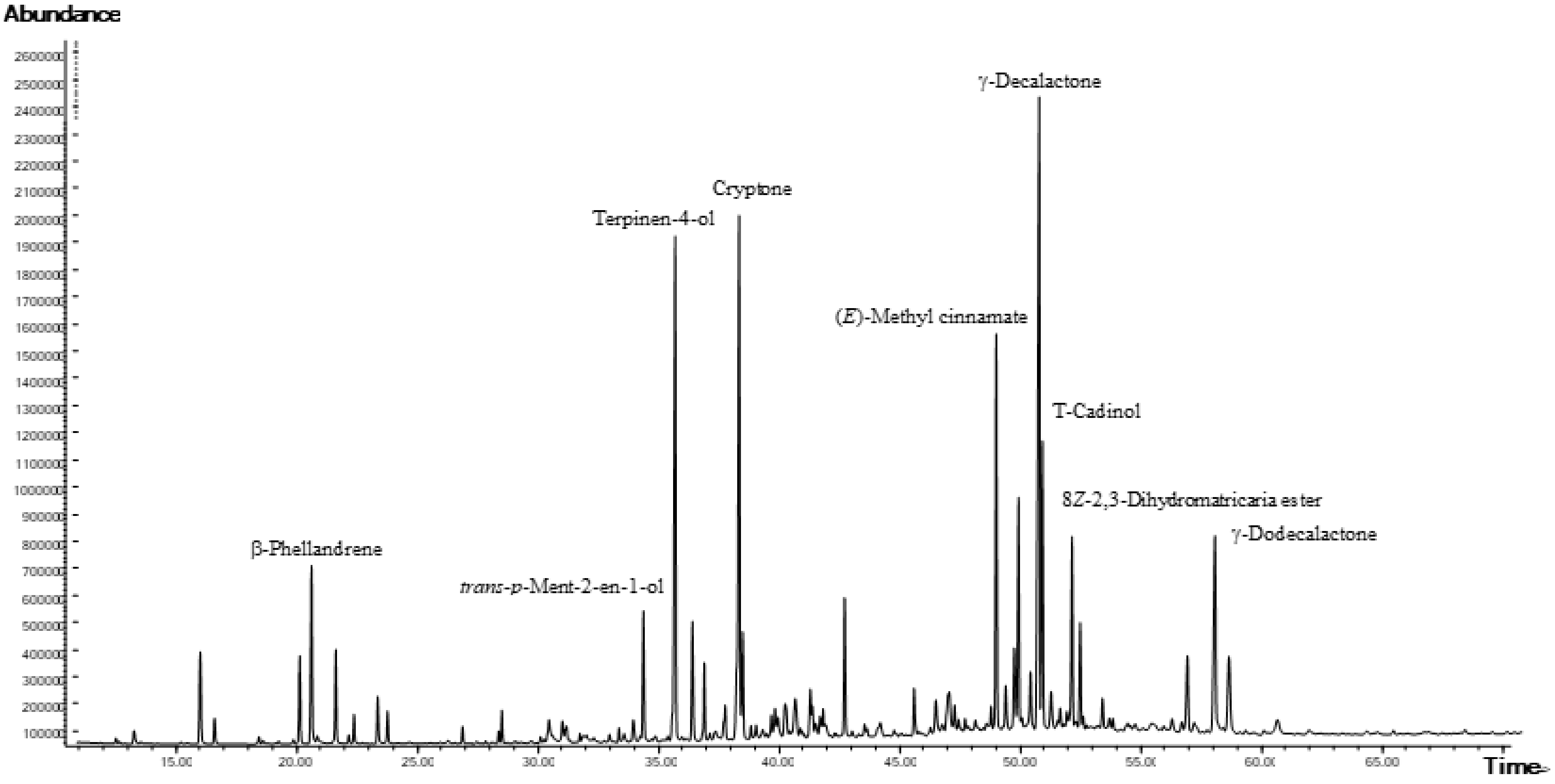

Supplementary Figure S1. Representative chromatogram of *E. nauseosa* essential oil analyzed using gas chromatography with FID detector.
